# Characterization of the Developing Lacunocanalicular Network During Fracture Repair

**DOI:** 10.1002/jbm4.10525

**Published:** 2021-07-12

**Authors:** Michele Casanova, Aaron Schindeler, Lauren Peacock, Lucinda Lee, Philipp Schneider, David G. Little, Ralph Müller

**Affiliations:** ^1^ Institute for Biomechanics ETH Zurich Zurich Switzerland; ^2^ Orthopaedic Research & Biotechnology The Children's Hospital at Westmead Westmead Australia; ^3^ Discipline of Child and Adolescent Health University of Sydney Camperdown Australia; ^4^ Bioengineering Science Research Group, Faculty of Engineering and Physical Sciences University of Southampton Southampton UK; ^5^ High‐Performance Vision Systems, Center for Vision, Automation & Control Austrian Institute of Technology (AIT) Vienna Austria

**Keywords:** BONE QUALITY, CANALICULI, OSTEOCYTE LACUNAE, LACUNOCANALICULAR NETWORK, FRACTURE REPAIR, MICRO–COMPUTED TOMOGRAPHY

## Abstract

Fracture repair is a normal physiological response to bone injury. During the process of bony callus formation, a lacunocanalicular network (LCN) is formed de novo that evolves with callus remodeling. Our aim was the longitudinal assessment of the development and evolution of the LCN during fracture repair. To this end, 45 adult wild‐type C57BL/6 mice underwent closed tibial fracture surgery. Fractured and intact contralateral tibias were harvested after 2, 3, and 6 weeks of bone healing (*n* = 15/group). High‐resolution micro–computed tomography (μCT) and deconvolution microscopy (DV) approaches were applied to quantify lacunar number density from the calluses and intact bone. On histological sections, Goldner's trichrome staining was used to assess lacunar occupancy, fluorescein isothiocyanate staining to visualize the canalicular network, and terminal deoxynucleotidyl transferase–mediated deoxyuridine triphosphate‐biotin nick end labeling (TUNEL) staining to examine osteocyte apoptosis. Analysis of μCT scans showed progressive decreases in mean lacuna volume over time (−27% 2–3 weeks; −13% 3–6 weeks). Lacunar number density increased considerably between 2 and 3 weeks (+156%). Correlation analysis was performed, showing a positive linear relationship between canalicular number density and trabecular thickness (*R* ^2^ = 0.56, *p* < 0.001) and an inverse relationship between mean lacuna volume and trabecular thickness (*R* ^2^ = 0.57, *p* < 0.001). Histology showed increases in canalicular number density over time (+22% 2–3 weeks, +51% 3–6 weeks). Lacunar occupancy in new bone of the callus was high (>90%), but the old cortical bone within the fracture site appeared necrotic as it underwent resorption. In conclusion, our data shows a progressive increase in the complexity of the LCN over time during fracture healing and demonstrates that this network is initiated during the early stages of repair. Further studies are needed to address the functional importance of osteocytes in bone healing, particularly in detecting and translating the effects of micromotion in the fracture. © 2021 The Authors. *JBMR Plus* published by Wiley Periodicals LLC on behalf of American Society for Bone and Mineral Research.

## 1 Introduction

Osteocytes are bone cells buried within the bone matrix that act as mechanotransducers^(^ ^1^ ^)^ and orchestrate bone remodeling.^(^ ^2^ ^)^ They communicate with each other via the lacunocanalicular network (LCN), which is critical for regulating bone homeostasis.^(^ ^2^ ^)^ The LCN has been recently shown to play a major role in the spatial distribution of mass density (ie, mineralization level) in bone^(^ ^3^, ^4^ ^)^ and acts to modulate bone mineral based on paracrine and hormonal factors.^(^ ^5^, ^6^ ^)^

As the LCN has a key role in modulating deposition, absorption, and mineralization of bone, it fundamentally influences the biomechanics of the bone matrix. Multiple studies have shown that osteocyte number density can be positively correlated with bone biomechanical properties and its resistance to fracture.^(^ ^7^, ^8^, ^9^, ^10^ ^)^ In fact, both lacunar number density and mean lacuna volume are correlated with the propagation of microcracks.^(^ ^10^, ^11^, ^12^ ^)^ It is established that lacunae can act as force concentrators,^(^ ^13^ ^)^ and can lead to bone fragility in poorly ordered bone structures.^(^ ^14^ ^)^ Furthermore, changes in the organization of canaliculi have been suggested to affect the mechanical properties of bone.^(^ ^15^, ^16^, ^17^ ^)^

During fracture repair, new woven bone is produced to bridge the fracture gap, and within this regenerating hard callus tissue a new LCN is required to form de novo. The importance of the forming LCN within bone healing has been a subject for recent discussion,^(^ ^18^ ^)^ and may have a central role in determining callus size.^(^ ^19^, ^20^ ^)^ Micromotion has been shown to be important for fracture healing, whereas overly rigid fixation can lead to stress shielding and insufficient new bone formation, which may be monitored by the nascent LCN.

In this preclinical study, we aimed to investigate how lacunar measures and canalicular number density evolve during fracture repair. We hypothesized that lacunar number and canalicular number density would change longitudinally during bone healing. Prior work demonstrated the formation of the LCN during bone healing and examined pharmacological modulation of the LCN, but these studies were limited to a single time point.^(^ ^21^ ^)^ Analysis was performed to investigate whether callus macroarchitecture and microarchitecture could be correlated to lacunar measures or canalicular number density, because these factors are related to bone deposition and structure in intact bone.^(^ ^19^, ^22^, ^23^, ^24^ ^)^ Although lacunar number density can be derived from micro–computed tomography (μCT) imaging, lacunar occupancy required further histological analysis. In particular, the presence of apoptotic osteocytes could possibly initiate perilacunar osteolysis and localized bone destruction.^(^ ^22^, ^25^ ^)^

On these grounds, we investigated the LCN development at three time points during murine bone fracture repair. Following closed tibial fracture surgery, the LCN was characterized using morphometric measures at 2‐week, 3‐week, and 6‐week time points after fracture. These represent distinct phases of the bone healing process. Two weeks represents an early stage of fracture repair, where the callus is mostly cartilaginous and the LCN is starting to be formed. Three weeks presents a consolidated callus, which is mostly mineralized and also around its peak in bone volume. Six weeks denotes a callus that is finalizing remodeling toward the original bone shape.

This study also features innovations in examining the LCN network. In prior studies, quantification of the LCN has been restricted to small regions encompassing a limited number of osteocyte lacunae and/or osteocytes.^(^ ^26^ ^)^ In this study, we have used an advanced approach combining deconvolution microscopy (DV) and high‐resolution μCT.^(^ ^27^ ^)^ This enabled us to quantify large regions from the central portion of the calluses, providing a broader overview on the LCN development in bone fracture repair. μCT was also performed on the whole calluses at lower spatial resolutions to compute tissue mineral density (TMD), bone volume fraction, and microarchitectural measures. Goldner's trichrome stain was performed on a subset of histological sample sections to assess lacunar occupancy and a terminal deoxynucleotidyl transferase–mediated deoxyuridine triphosphate‐biotin nick end labeling (TUNEL) stain was used to examine apoptosis. Together, these imaging modalities reveal a complex picture of the longitudinal changes in the LCN that occur over time during fracture healing.

## 2 Materials and Methods

### 2.1 Animals and surgery

Forty‐five female, 11‐week‐old wild‐type mice (C57BL/6) were used in this study. All animals underwent a closed fracture on their right tibia using an established fracture protocol.^(^ ^28^ ^)^ In brief, animals were anesthetized with ketamine and xylazine and a complete fracture was made without breaking the skin using a handheld device made from surgical staple removers. Fractures were fixed via the intramedullary canal with solid stainless‐steel insect pins inserted via the knee. Fractures were confirmed using X‐ray radiography and animals were monitored by weekly X‐ray radiographies during the fracture healing process (Faxitron MX‐20; Faxitron, Tucson, AZ, USA). Animals were given buprenorphine (0.05 mg/kg) for analgesia up to every 12 hours as required. Fractured mice were subsequently harvested at three different time points (*n* = 15 per time point). Group 1 was harvested 2 weeks after fracture, group 2 after 3 weeks, and group 3 after 6 weeks. All animal experiments were approved by the Westmead Hospital Animal Ethics Committee.

### 2.2 Specimen collection

Fractured and contralateral tibias were extracted and fixed in 4% phosphate‐buffered formalin overnight at 4°C. After fixation, the samples were immersed in 30% sucrose solution for a day, then snap frozen in optimum cutting temperature (OCT) medium (TissueTek OCT Compound; Thermo Fisher Scientific, Waltham, MA, USA) and cryosectioned (Leica CM1950; Leica, Wetzlar, Germany). The bones were cut in the sagittal plane, starting from the medial side. Sections of 5, 7, and 20 μm thickness were cut close to the central portion, paying attention to conserve the lateral part of the callus intact for subsequent μCT. Sections were taken using cryofilm to preserve the intact bone (SECTION‐LAB Co. Ltd., Hiroshima, Japan). The cryofilm sections were adhered to glass microscope slides using a chitosan adhesive (1% Chitosan; MilliporeSigma, St. Louis, MO, USA; in 0.25% acetic acid) and left to dry at 4°C overnight.

### 2.3 μCT

μCT scans and morphometric analyses were performed according to published methods for the assessment of bone microstructure using μCT.^(^ ^29^ ^)^ To investigate lacunar number density and mean lacuna volume, all lateral portions of the 90 samples were scanned using a μCT system (μCT 50; Scanco Medical, Brüttisellen, Switzerland). Only lateral portions could be scanned because samples were cryosectioned after extraction (see Specimen collection section, above). A stack with a height of 1.1 mm was acquired from the central portion of the calluses and from the nonfractured tibias at 45% of the tibial length starting distally. An isotropic voxel size of 1.2 μm, a tube voltage of 70 kVp, an X‐ray intensity of 57 μA and an integration time of 1.5 seconds were selected. A Gaussian filter (sigma = 0.8, support = 1.0) was applied for noise reduction. Old bone and newly formed bone were segmented using customized Image Processing Language (IPL) file scripts (Scanco Medical). Lacunar number density (defined as number of lacunae to bone volume + lacunar porosities) and mean lacuna volume (defined as total lacunar volume divided by number of lacunae) were inferred from both old and newly formed bone.

To assess callus bone volume fraction, TMD, and microarchitectural measures, all bone calluses were scanned again including all newly formed bone using μCT. An isotropic voxel size of 7 μm, a tube voltage of 55 kVp, an X‐ray intensity of 72 μA, and an integration time of 1.5 seconds were set. A Gaussian filter (sigma = 0.8, support = 1.0) was applied for noise reduction. We then used IPL to manually segment the newly formed bone, excluding the bone in the intramedullary canal. Regions in which the porosity was inferior to 50% of the local total volume were considered dense woven bone and excluded for further analysis. Bone volume fraction was then assessed in these subvolumes (macroarchitectural measure). For computing the microarchitectural measures, the microstructure of the mineralized struts of the calluses was then isolated from the dense woven bone. A bone callus of the 6‐week postoperative (post‐op) group was removed from the subsequent computations for the absence of a significant quantity of struts (sample toward the end of the remodeling phase). Trabecular number (Tb.N), trabecular thickness (Tb.Th), trabecular separation (Tb.Sp), standard deviation of Tb.Th (Tb.Th.SD), standard deviation of Tb.Sp (Tb.Sp.SD), degree of anisotropy (DA), connectivity density (Conn.D), and the structure model index (SMI) were then computed as described by Bouxsein et al.^(^ ^29^ ^)^

### 2.4 Canalicular number density

Four 20‐μm‐thick sections of each sample were used to investigate canalicular number density. The samples were dehydrated, then covered with a 1% fluorescein isothiocyanate (FITC) solution in absolute alcohol and left overnight at 4°C. The samples were mounted and coverslipped, then analyzed using a deconvolution microscope (Deltavision, Isaquah, WA, USA). Datasets of 60 stacks with a thickness of 0.2 μm and a magnification of ×60 were acquired. In each of the 90 samples, the cell processes of 10 to 12 osteocytes were examined. The osteocytes analyzed were in the central portion of the calluses and in intact tibias at the 45% of the tibial length starting distally, which corresponds to the height where the fracture was created in the opposite tibia (fractured tibia). To estimate canalicular number density, a region of interest (ROI) was defined by contouring the lacuna on the optical section with its largest visible area. Because osteocyte processes fan out radially, the upper limit of the imaged ROI was then translated ~1 μm above the cell body. The canaliculi in the ROIs were then manually counted using ImageJ (U.S. National Institutes of Health Bethesda, MD, USA; https://imagej.nih.gov/ij/) and reported relative to the ROI size to calculate the two‐dimensional (2D) canalicular density. To facilitate their identification, edge enhancement and absolute thresholding of the images was performed.

### 2.5 Lacunar occupancy and histological assessment

Two 5‐μm‐thick sections from five fractured tibias per group and their respective contralateral bones were stained with Goldner's trichrome. Osteocyte occupancy was assessed in both newly formed and intact bone. To determine osteocyte occupancy, 10 subregions covering a total bone area of 1 mm^2^ were selected for each sample. The subregions were sampled from the central portion of the calluses and on the cortical bone of the intact tibias at the 45% of the tibial length starting distally. Empty lacunae were defined as lacunae without any visible remnant of cellular material. To prevent false negatives, only lacunae with a visible cross‐section of at least 16 μm^2^ were considered in the assessment.

A 7‐μm‐thick tibial fracture section from each mouse in each group (*n* = 15) was stained using the DeadEnd™ Colorimetric TUNEL System (Promega Corporation, Madison, WI, USA) to label apoptotic cells in the fracture callus with diaminobenzidine (DAB). The standard protocol for this kit was followed. Tissue sections were counterstained with Harris' hematoxylin (POCD Scientific, North Rocks, Australia), mounted with Aquatex (Merck Group, Darmstadt, Germany) and coverslipped for imaging on an Aperio Scanscope brightfield slide scanner (Leica Biosystems, Mt Waverley, Australia) using the 40× objective. Representative images from each group were selected.

### 2.6 Statistical analysis

All statistical analyses were performed using SPSS Statistics (version 20; IBM, Armonk, NY, USA). Mean and standard deviation (SD) were given for all the results. One‐way analysis of variance (ANOVA) with Bonferroni post hoc test was used for the analysis comparing the three groups representing different stage of bone fracture repair. For comparisons between bone calluses and intact tibias, paired Student's *t* tests were performed. For investigating possible correlations between morphometric measures, Pearson product–moment and quadratic correlation coefficients were computed. Paired Student's *t* tests were used for the comparisons between mean lacuna volume of necrotic bone and of intact tibias in the three different time points. For all analyses, *p* ≤ 0.05 was considered to indicate statistical significance.

## 3 Results

### 3.1 Animals and surgery

No unexpected or adverse events occurred during the surgery procedure nor during postoperative monitoring. No specimens were excluded from analysis.

### 3.2 μCT

Bone volume fraction, TMD, microarchitectural measures, and lacunar measures were estimated for the callus region at 2 weeks, 3 weeks and 6 weeks postoperatively and compared with intact contralateral bone (Table 1). This well‐described fracture model^(^ ^28^ ^)^ shows endochondral ossification of the soft callus at ~2 weeks, robust woven bone callus at ~3 weeks, and substantive new cortical bone remodeling by ~6 weeks. As healing progresses, the fracture restores itself to resemble the contralateral nonfractured bone.

**Table 1:** Quantitative μCT Macro/Microarchitecture, Lacunar Occupancy, and Tissue Mineral Density of the Bone Calluses (New Bone) and Intact Tibias

Parameter	2 weeks postoperation	3 weeks postoperation	6 weeks postoperation
Lacunar and canalicular measures, mean ± SD			
Bone calluses	*n* = 15	*n* = 15	*n* = 15
Ca.D (1/μm^2^)	0.082 ± 0.021	0.10 ± 0.033*	0.151 ± 0.033*** ^,^ *****
<Lc.V> (μm^3^)	501 ± 99	366 ± 29***	319 ± 27*** ^,^ ****
N.Lc/BV (10^3^/mm^3^)	20.4 ± 4.3	52.4 ± 11.5***	42.9 ± 7.5*** ^,^ ****
Lacunar occupancy (%)	94 ± 3.5 (*n* = 5)	94 ± 2.8 (*n* = 5)	94 ± 1.5 (*n* = 5)
Intact tibias	*n* = 15	*n* = 15	*n* = 15
Ca.D (1/μm^2^)	0.179 ± 0.035	0.175 ± 0.034	0.182 ± 0.039
<Lc.V > (μm^3^)	302 ± 34	297 ± 29	312 ± 26
N.Lc/BV (10^3^/mm^3^)	52.7 ± 13.4	44.9 ± 11.7	47 ± 11.5
Lacunar occupancy (%)	93 ± 3.2 (*n* = 5)	92 ± 0.9 (*n* = 5)	90 ± 3.9 (*n* = 5)
Macroscopic measures, mean ± SD			
Bone calluses	*n* = 15	*n* = 15	*n* = 15
BV/TV (%)	36.5 ± 6.0	43.9 ± 8.4*	37.3 ± 9.6
TMD (HA mg/cm^3^)	627 ± 44	796 ± 26***	920 ± 24*** ^,^ *****
Microarchitectural measures, mean ± SD			
Bone calluses	*n* = 15	*n* = 15	*n* = 14
Tb.N (1/mm)	12.8 ± 1.6	7.8 ± 1.5***	3.3 ± 0.7*** ^,^ *****
Tb.Th (mm)	0.034 ± 0.0025	0.049 ± 0.0053***	0.085 ± 0.0159*** ^,^ *****
Tb.Sp (mm)	0.072 ± 0.0129	0.124 ± 0.0251***	0.300 ± 0.0526*** ^,^ *****
Tb.Th.SD (mm)	0.0121 ± 0.0016	0.0196 ± 0.0058*	0.0386 ± 0.0117*** ^,^ *****
Tb.Sp.SD (mm)	0.479 ± 0.020	0.072 ± 0.024*	0.130 ± 0.031*** ^,^ *****
DA (−)	1.059 ± 0.014	1.213 ± 0.081***	1.543 ± 0.140*** ^,^ *****
Conn.D (1/mm^3^)	3753 ± 524	1537 ± 378***	93 ± 58*** ^,^ *****
SMI (−)	0.895 ± 0.350	0.484 ± 0.659	1.870 ± 0.756*** ^,^ *****

μCT = micro–computed tomography; <Lc.V> = mean lacuna volume; BV/TV = bone volume fraction; Ca.D = canalicular number density; Conn.D = connectivity density; DA = degree of anisotropy; HA, hydroxyapatite; N.Lc/BV = lacunar number density; SD = standard deviation; SMI = structure model index; Tb.N = trabecular number; Tb.Sp.SD = standard deviation of Tb.Sp; Tb.Sp = trabecular separation; Tb.Th.SD = standard deviation of Tb.Th; Tb.Th = trabecular thickness; TMD = tissue mineral density.

* *p* < 0.05 when compared to 2 weeks postoperation.

** *p* < 0.01 when compared to 2 weeks postoperation.

*** *p* < 0.001 when compared to 2 weeks postoperation.

**** *p* < 0.05 when compared to 3 weeks postoperation.

***** *p* < 0.001 when compared to 3 weeks postoperation.

**Fig 1:**
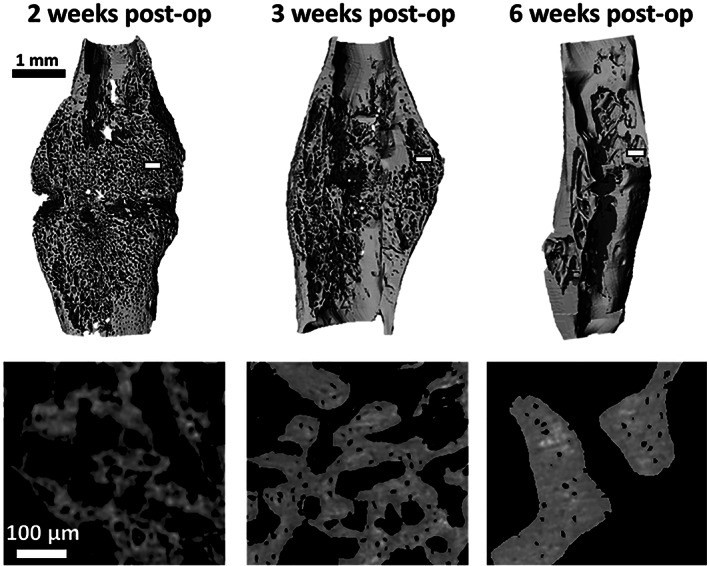
Three‐dimensional rendering of representative calluses (scans at 7 μm voxel size) for each time point (top). Representative details of the callus struts from the transverse plane (scans at 1.2 μm voxel size) at the three post‐op time points; acquired in the white rectangular area on the top images (bottom). post‐op = postoperation.

Representative details of binarized high‐resolution μCT scans for each group and representative three‐dimensional renderings of the calluses for each group are presented in Fig. 1. Mean lacuna volume significantly decreased by 27% from 2 weeks to 3 weeks post‐op (*p* < 0.001). Between 3 and 6 weeks post‐op, the decrease was only 13% (*p* < 0.05). Lacunar number density was found to be relatively low at 2 weeks, but significantly increased by 156% at 3 weeks (*p* < 0.001) before decreasing by 18% (*p* < 0.05) at 6 weeks. A paired Student's *t* test detected a significant lower lacunar number density in the 6‐weeks calluses compared to their respective contralateral tibias.

**Fig 2:**
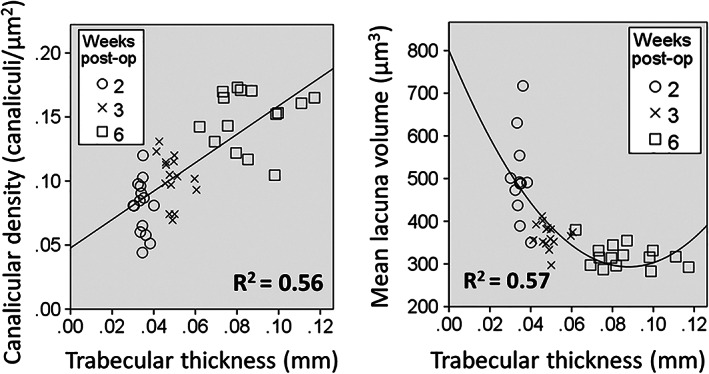
Trabecular thickness versus canalicular number density or lacunar volume for all calluses of the three time points: 2, 3, and 6 weeks postoperatively. A significant linear correlation of *R* ^2^ = 0.56 (*p* < 0.001) was found for canalicular number density, and a significant quadratic correlation of *R* ^2^ = 0.57 (*p* < 0.001) was found for mean lacuna volume. post‐op = postoperation.

To investigate if LCN morphology is linked to the architecture of the callus, correlations (linear, quadratic, cubic) between macroarchitecture and microarchitectural measures and LCN measures (canalicular number density, lacunar number density, mean lacuna volume) were calculated within the group and versus all groups pooled. Relationships between calluses and intact tibias were also analyzed. Table 2 presents the results of the linear correlation analysis, and statistically significant correlations were observed when data from all time points were pooled (Fig. 2). In the early stages of the callus development, mean lacuna volume has a high relative SD, but a constant trabecular thickness; in a later stage of fracture repair, lacuna volume is relatively stable around 320 μm^3^, but trabecular thickness varies considerably.

**Table 2:** Statistically Significant Pearson Correlations Between Microarchitectural Measures and Lacunocanalicular Measures

Parameter	Tb.Th.SD ~ Lc.D (*n* = 15)	DA ~ Lc.D (*n* = 15)	Tb.N ~ Ca.D (*n* = 15)	Tb.Sp ~ Ca.D (*n* = 15)	Conn.D ~ Ca.D (*n* = 15)	SMI ~ Ca.D (*n* = 14)
Postoperative week	2	3	3	3	3	6
Pearson's *R*	0.738	0.708	0.709	−0.808	0.753	0.706
*p*	0.006	0.003	0.003	0.000	0.001	0.003

Ca.D = canalicular number density; Conn.D = connectivity density; DA= degree of anisotropy; Lc.D = lacunar number density; SMI = structure model index; Tb.N = trabecular number; Tb.Sp = trabecular spacing; Tb.Th.SD = standard deviation of Tb.Th.

### 3.3 Canalicular number density, lacunar occupancy, and osteocyte apoptosis

**Fig 3:**
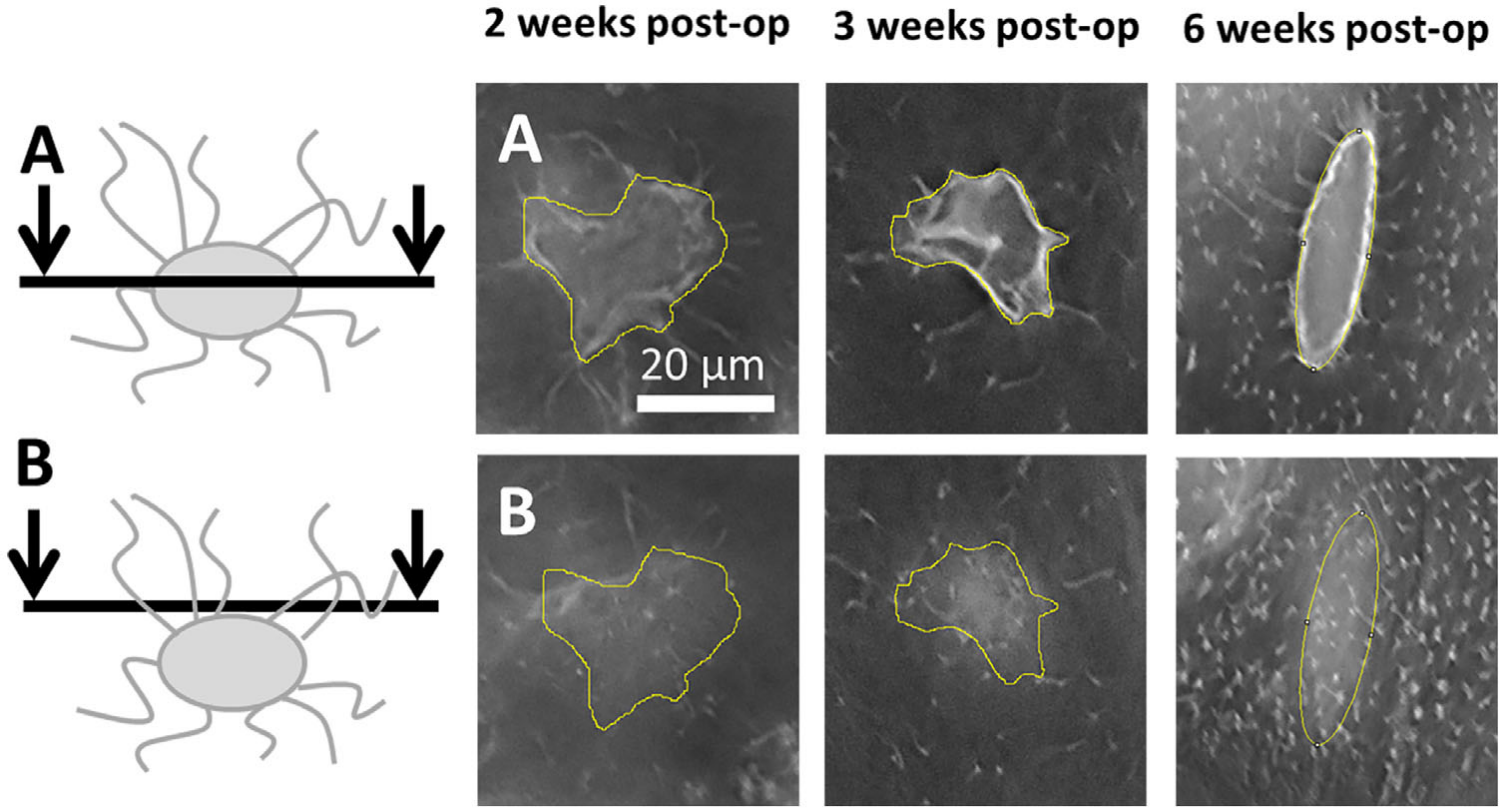
Illustrative images of canaliculi in the calluses at the three different time points. The yellow area delimits the lacunar contour (ROI). (*A*) Microscopy sections cutting through the center of the lacunae. (*B*) Confocal planes placed a few micrometers above the lacunae. post‐op = postoperation; ROI = region of interest.

Representative images for canalicular number density are shown in Fig. 3A,B. At all stages of callus formation and remodeling, canalicular number density was less than that seen in intact bone yet increasing over time. The results for canalicular number density are presented in Table 1. Paired Student's *t* tests revealed statistically significant differences (*p* < 0.001) between calluses and contralateral tibias for all three time points. Analysis of the calluses between the different time points by one‐way ANOVA showed a significant difference. Post hoc analysis revealed a +22% increase in canalicular number density (*p* < 0.05) between weeks 2 and 3. There was a +51% increase in canalicular number density (*p* < 0.001) between weeks 3 and 6.

**Fig 4:**
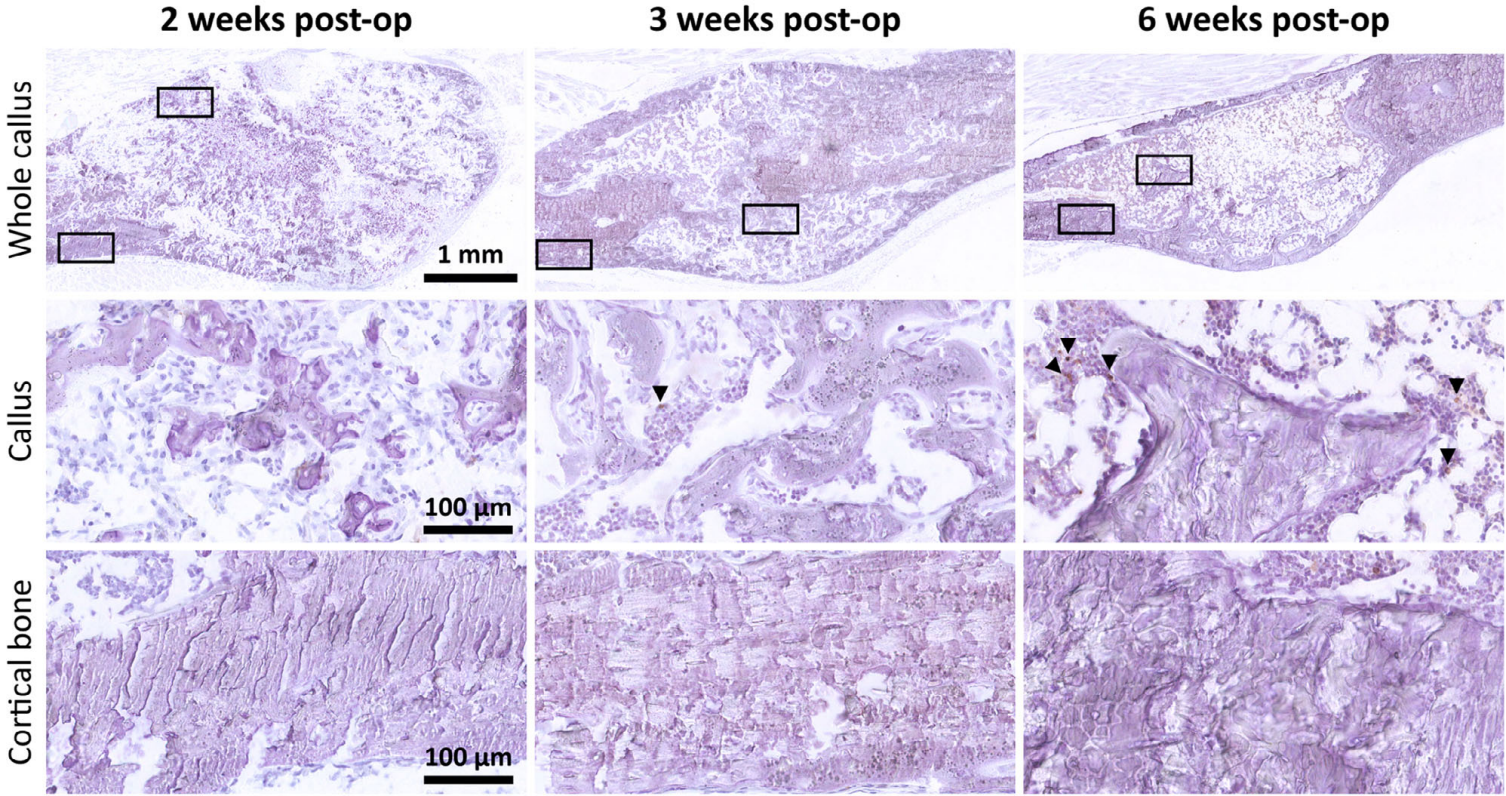
TUNEL staining of fracture calluses (top row) with 40× magnified regions below (boxed) corresponding to the callus tissue (middle row) and periosteal cortical bone adjacent to the callus (bottom row). TUNEL staining (brown), hematoxylin counterstain (purple). post‐op = postoperation; TUNEL = terminal deoxynucleotidyl transferase–mediated deoxyuridine triphosphate‐biotin nick‐end labeling.

TUNEL staining showed a very low level of apoptotic cells at all time points (Fig. 4). No apoptotic cells were observed in the parosteal cortex adjacent to the callus. Within the callus itself, few apoptotic cells were noted and were consistent with non‐bone cells in the marrow space rather than osteoblasts/osteocytes. The tissue section quality was poorer in thin sections featuring regions of necrotic bone compared to the thicker sections used for quantification of the canaliculi.

**Fig 5:**
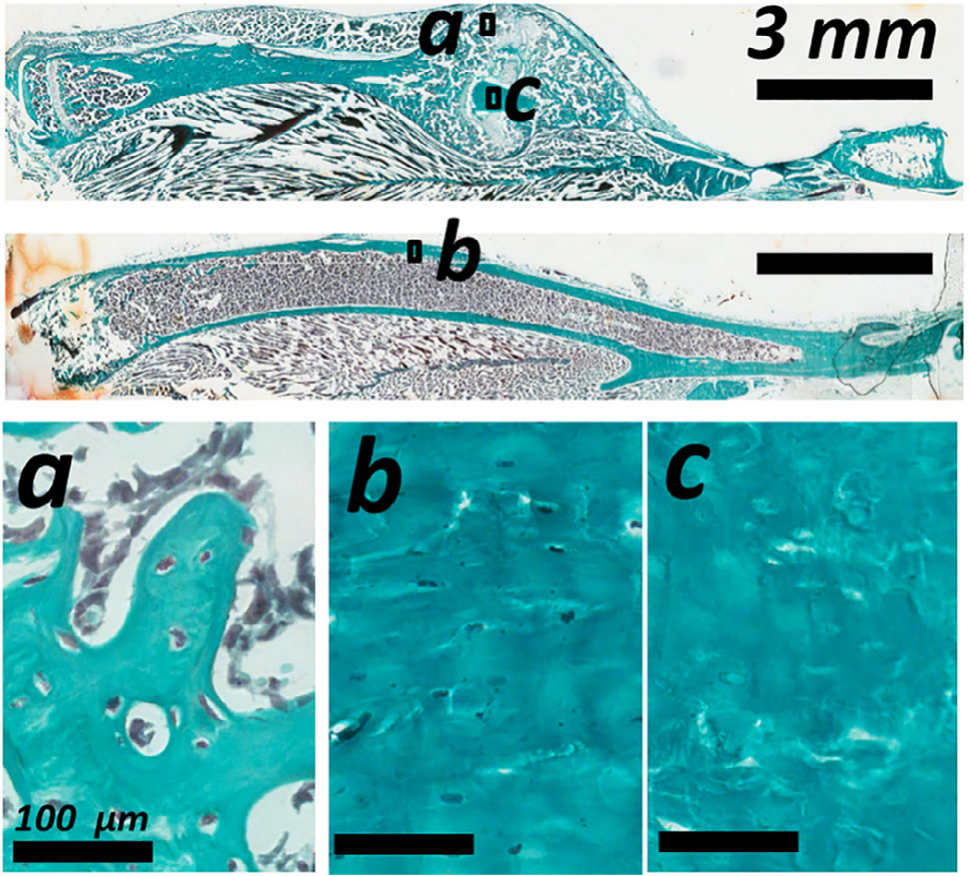
Descriptive histology with Goldner's trichrome staining showing lacunar occupancy in the woven bone of (*a*) the bone callus, (*b*) the cortical bone of the intact tibia, and (*c*) on the reabsorbing cortical bone in the bone callus.

Lacunar occupancy was high in the new callus and the old intact cortical bone of the tibia (Fig. 5*a,b*, respectively), exceeding 90%. No significant difference in lacunar occupancy was detected, neither between the time points nor between calluses and intact bones. Within the callus some regions of old fractured cortical bone were present and showed evidence of necrosis and loss of lacunar occupancy (Fig. 5*c*). No remnant cellular material was visible. These regions were further analyzed.

### 3.4 Characterization of resorbing old cortical bone

**Fig 6:**
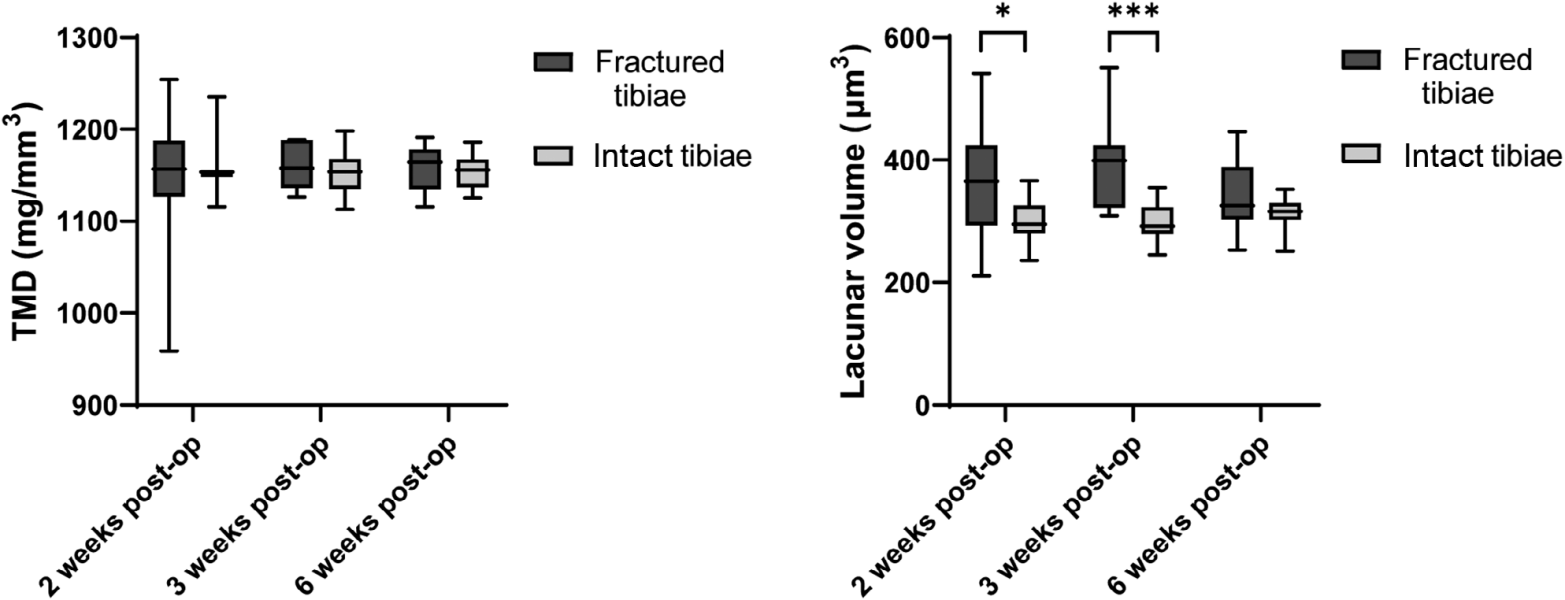
TMD of the necrotic bone in the bone calluses (fractured tibias) compared to the TMD of the intact tibias showed no significant difference. However, the mean lacuna volume of necrotic (dead) old cortical bone was significantly higher than other bone at the 2‐week and 3‐week time points. Bars represent standard deviation. *p < 0.05; **p < 0.01. post‐op = postoperation; TMD = tissue mineral density.

From the sections stained with Goldner's trichrome, regions corresponding to the old cortex were identified as having empty osteocyte lacunae (ie, 0% lacunar occupancy). These areas were regions selected from 10 to 11 of the higher resolution μCT scans (1.2 μm voxel size) of the calluses per group and contained at least 100 lacunae. To confirm that the regions considered were not from new bone deposition, TMD (excluding canals and lacunar porosities) of the subvolumes was compared to TMD of the scans of the intact tibias. A plot of TMD of necrotic bone of the three groups and all contralateral tibias showed no statistically significant difference between TMD of the calluses and TMD of the intact tibias (Fig. 6). Comparisons between mean lacuna volume of necrotic bone and of intact tibias in the three different groups showed significant differences in mean lacuna volume between regions with necrotic cortical bone in the calluses and the intact tibias 2 weeks post‐op (+20%, *p* < 0.05) and 3 weeks post‐op (+27%, *p* < 0.001) (Fig. 6).

## 4 Discussion

μCT is a versatile imaging technique that has become the gold standard for preclinical bone analysis. Historically, the quality of μCT images precluded the analysis of osteocyte lacuna; however, improvements seen with μCT enable osteocyte lacuna to be visualized. Other groups have highlighted technical advances in μCT imaging in bone in recent years. Longitudinal μCT has been performed to capture the healing process of bone healing^(^ ^30^ ^)^; however, in vivo imaging systems typically have far poorer spatial resolutions than those used with harvested specimens, mainly because of the limited X‐ray dose employed for the animals scanned in vivo.

Although hardware capabilities have a considerable impact on the data that can be collected by μCT, the importance of software and computational analysis is often underrated. In this study, we used a Gaussian filter to remove noise and used custom scripts to identify mineralized bone. For some quantitative analysis, manual segmentation was performed as automated segmentation of fractures can be particularly challenging. Although there have been efforts to develop programs that enable accurate automated μCT segmentation,^(^ ^31^ ^)^ to date they remain less accurate than manual approaches.

μCT can increase in power when combined with other scanning and analysis modalities. For example, prior studies have attempted to combine μCT with laser Doppler to characterize neovascularization in healing fractures.^(^ ^32^ ^)^ Our results present comprehensive snapshots of LCN development in a quantitative fashion and were enabled by histological analysis combined with deconvolution microscopy/μCT. Our imaging process with DV microscopy offered an excellent lateral resolution, nevertheless axial resolution did not allow us to precisely quantify canalicular expression in radial direction. Thus, canalicular number density was computed on the longitudinal plane of the bone. In cortical bone of the mouse femur, canalicular number density has been shown to be higher in the radial direction than in the tangential direction.^(^ ^33^ ^)^

This work's findings are consistent with genetically modified mouse models that examine osteocytes and osteocyte gene expression in the callus. For example, conditional deletion of *Igf1* using a *Dmp1‐cre* transgene accelerated bony union in mice.^(^ ^34^ ^)^ This and other publications employing osteocyte‐targeted knockout models have implied a key role for osteocyte signaling within callus. However, these studies must be interpreted within the context of off‐target gene deletion by *Dmp1‐cre* in other cell lineages as detected using more sensitive reporters.^(^ ^35^ ^)^ Hence, our study showing the temporal development of the LCN will be useful for clarifying prior genetic mouse models.

This study identified two types of bone within the callus region. First, there was the new mineralization of the woven bone callus that rapidly establishes an LCN. Correlation analysis indicates that this network grows in complexity as the callus remodels and while the osteocyte lacuna decreases in size, the occupancy remains high. In contrast, the bone of the former cortex become necrotic, typified by a loss of osteocytes, and will resorb over time as the new cortex forms. New bone versus old bone was also represented by an increased TMD in the old cortex. By 6 weeks, the fracture callus was highly remodeled.

This study has several limitations. Although it assessed several time points, there is a fundamental variability in the fracture healing process such that some fractures may progress faster than others. Still, this is superior to prior studies by our group that examined on a single time point.^(^ ^21^ ^)^ Group sizes were suitable for assessing most of the primary outcome measures by μCT, but it is likely underpowered for intergroup (time point) linear correlation analysis. Nevertheless, when groups were pooled, statistically significant correlations were observed. Finally, threshold selection was determined based on arbitrary density cutoffs and thus lacuna volume and lacunar number density data should be considered in terms of differences between groups and changes over time, rather than as empirical values. Variations in mineral density in the different portion of the calcified callus encouraged us to try a gradient‐threshold edge detection method; however, this method was unable to produce consistent results.

From a biological standpoint, the formation of the LCN is likely mediated by enzymatic degradation of the bone matrix by matrix metalloproteases (MMPs). MMP2 is necessary for LCN formation based on the reported knockout mouse phenotype^(^ ^36^ ^)^ and critical for fracture remodeling.^(^ ^37^ ^)^ MMP‐9 knockout mice show impaired fracture repair, but the LCN was not specifically examined in this model.^(^ ^38^ ^)^ MMP‐13 is needed for perilacunar remodeling as well as maintaining a normal canalicular network,^(^ ^39^ ^)^ yet its role in fracture repair remains unclear.

Further research will be needed to assess the functional importance of MMPs as well as other factors expressed by osteocytes. Osteocytes express a range of important regulatory secreted proteins, such as *Sclerostin* and *RankL*, which modulate local bone formation and resorption. Thus, osteocytes and their secretome may be important for the regulation of the fracture healing process. Use of preclinical models that modulate the LCN using drugs^(^ ^21^ ^)^ or genetic manipulation will enable the role of the LCN to be more clearly elucidated. The interaction between the nascent LCN and revascularization or bone biomechanics are also areas where there is scope for future studies. Deficiencies in blood supply are associated with impaired bone healing. Loss of stability during fracture healing can result in abundant bony callus formation with impaired union (ie, hypertrophic non‐union), and it is possible that the LCN may be critical for detecting stability and transducing signals.

In conclusion, this study is the first to present a detailed analysis of the development of the LCN during fracture repair. We identified significant increases in canalicular number density as bone repair progressed, whereas mean lacuna volume significantly decreases over this time period. This study illustrates the advantage of using multiple X‐ray and classical histological imaging modalities to describe the formation and evolution of a new LCN in bone seen within the fracture callus.

## Author Contributions

**Michele Casanova:** Data curation; formal analysis; investigation; methodology; validation; writing ‐ original draft; writing‐review & editing. **Lauren Peacock:** Investigation; methodology; writing‐review & editing. **Lucinda Lee:** Formal analysis; investigation; methodology; writing‐review & editing. **Philipp Schneider:** Methodology; supervision. **David Little:** Conceptualization; funding acquisition; investigation; supervision; writing‐review & editing.

## Conflict of interest

The authors declare no conflict of interest.

5

### Peer Review

The peer review history for this article is available at https://publons.com/publon/10.1002/jbm4.10525.
